# To test or not to test? Study protocol for a best-worst scaling to understand decision-making and preferences for genetic testing in moderate-risk individuals

**DOI:** 10.1371/journal.pone.0339696

**Published:** 2025-12-29

**Authors:** Carina Oedingen, Nicolle Hua, Karen V. MacDonald, Julien Marcadier, Renee Perrier, Lindsay Tuer, Brenda McInnes, Francois Bernier, Deborah A. Marshall

**Affiliations:** 1 Department of Community Health Sciences, Cumming School of Medicine, University of Calgary, Calgary, Alberta, Canada; 2 Department of Medical Genetics, Cumming School of Medicine, University of Calgary, Calgary, Alberta, Canada; 3 Alberta Children’s Hospital Research Institute, Calgary, Alberta, Canada; International Medical University, MALAYSIA

## Abstract

**Introduction:**

Genetic testing is usually offered to individuals at high risk of carrying disease-causing variants. For those at moderate risk of genetic conditions, testing could also help in early detection, prevention, and treatment. Although individuals’ preferences to undergo genetic testing can influence their treatment decisions, there is limited research on preferences of moderate-risk individuals. This study aims to estimate the relative importance of factors that influence decision-making for genetic testing of moderate-risk individuals from different disease cohorts and testing types.

**Methods:**

We outline the study protocol for a best-worst scaling (BWS) object case (Case 1) and a ranking exercise around primary genetic testing and secondary analyses, respectively. Individuals (n = 350) at moderate risk of breast cancer or aortic disease will be recruited through genetic clinics who are part of PreventGene to complete an online preferences survey after deciding whether to have genetic testing, but before receiving the test results. Thirteen BWS items were selected based on the results of a scoping review and input from clinical experts. A balanced incomplete block design will be used. Respondents are asked to select the most (best) and least (worst) important factors in their decision-making. Data will be analysed using count analysis, multinomial logit, and latent class analyses. The data collection started in March 2025 and is expected to be finished by spring 2026.

**Discussion:**

Understanding how individuals at moderate risk make genetic testing decisions can help to better understand the decision-making process about what testing types should be available in which contexts and for which individuals. Findings can inform clinical and health policy decision-makers in planning and offering additional future genetic testing programs for moderate-risk individuals. The study is registered in the Open Science Framework (10.17605/OSF.IO/JFPH9).

## 1. Introduction

Genetic testing can help detect disease-causing variants, assess individuals’ and families’ risk of developing certain diseases, diagnose genetic conditions, and guide treatment decisions [1,2]. Genetic testing can be categorized by the purpose of different testing, including primary and secondary genetic testing. Primary genetic testing aims to identify genetic variants related to the primary reason for testing, while secondary genetic testing (also called secondary analyses) aims to intentionally identify genetic variants of actionable conditions unrelated to the primary concerns [3,4]. The uptake of these testing types in the clinical setting has increased rapidly over recent years because of its integral role in precision medicine and patients’ decision-making process [4–6].

Genetic testing is typically indicated for only individuals who are highly likely to have a disease-causing genetic variant (‘high-risk’) because of the low likelihood of detecting such variants in lower-risk population groups [7]. In Canada, for example, only individuals who satisfy certain eligibility criteria are considered as high-risk and are offered clinical genetic testing covered by the healthcare system [8–10]. Individuals with a personal and/or family history for the disease, yet do not satisfy the criteria or display classic ‘high-risk’ patterns, are otherwise considered moderately likely to carry a disease-causing genetic variant (‘moderate-risk’) [11]. Moderate-risk cohorts are typically ineligible for routine clinical genetic testing. They are also underrepresented in research as high-risk cohorts are more commonly recruited [12–14]. Examples of moderate-risk population groups for hereditary genetic diseases are breast cancer and aortic disease. Breast cancer is one of the most common causes of female cancer morbidity and mortality [15], and individuals at moderate risk have an increased lifetime risk of between 17–30% of developing breast cancer compared to the low-risk general population [16]. Aortic disease is also a common cause of death globally [17]. There is evidence of other underlying genetic factors based on the familial clustering studies on aortic size and dilation [18,19], which could develop into an aortic aneurysms and subsequent aortic dissection if left undetected [20]. While aortic disease is more prevalent in those with an underlying familial predisposition relative to the general population [18], the complexities of its genotype-phenotype correlation could result in clinical and familial presentations that are otherwise less obvious. This can lead to a potential risk for individuals who do not exhibit the classical high-risk indicators for hereditary aortic disease to be incorrectly assessed as ineligible for clinical genetic testing.

Accepting or declining genetic testing is complicated by life-changing psychological, social, and economic implications [21–23]. Individuals need to weigh the potential benefit-risk trade-offs in their decision, making it important to understand individual’s attitudes and preferences, especially with moderate-risk individuals. Current studies have explored stated preferences around genetic testing, either before individuals decide whether to have genetic testing, or after they already receive their genetic testing results [24–26]. A few studies also focused on general population preferences with unknown risk factors [27,28]. Relevant factors included testing types and performance, testing procedures and processes, personal factors, potential benefits and risks, as well as costs and willingness to pay [23,27–30].

The current state of the knowledge in genetic testing highlights the need to assess genetic testing preferences for individuals at moderate risk, as well as the timing of these assessments, to inform and optimize patient-centered care. Research in moderate-risk disease cohorts (i.e., breast cancer, aortic disease) will allow us to better understand preferences and uptake of genetic testing, and in turn, inform decision-making related to testing eligibility criteria and accessibility.

### 1.2. Study aims

This study is part of a larger project entitled ‘Precision Medicine Expansion to Moderate Risk Patients: Shaping the Future of Adult Precision Health Genomics (PreventGene)’. PreventGene aims to establish an integrated clinical and academic Adult Precision Health Genomics Program for individuals at moderate risk of breast cancer or aortic disease in Alberta, Canada. Additionally, stated preferences around genomic testing in individuals at moderate risk of genetic diseases will be elicited to develop and implement sustainable, evidence-informed clinical and screening programs for disease prevention in moderate-risk populations. The data collection started in March 2025 and is ongoing. In this sub-study, we will quantitatively estimate the relative importance of factors that influence individuals’ decision-making around genetic testing. The study aims are:

1. To elicit preferences (relative importance of factors that influence decision-making) of individuals at moderate risk of breast cancer or aortic disease for primary genetic testing and secondary analyses;
2. To compare preferences between individuals who chose to have primary genetic testing and/or secondary analyses to those who chose not to have them; and
3. To identify and understand any heterogeneity in preferences based on demographic characteristics and attitudes, as well as experiences towards genetic testing in general.

The study is registered on the Open Science Framework (10.17605/OSF.IO/JFPH9).

## 2. Materials and methods

### 2.1. Study design

A case 1 (object case) best-worst scaling (BWS) experiment will be used to elicit the relative importance/prioritisation of factors that influence decision-making for primary genetic testing (study aim 1). BWS is an increasingly used stated preference elicitation method [31] that is grounded in the random utility theory [32], resulting in utility maximization when choosing between pairwise options. It assumes that the utility an individual derives from a good or service can be divided into a systematic (predictable) component, and a random (unexplained) component and that individuals choose the option that provides the highest overall utility for them. This theory is the most widely accepted framework for analyzing stated choice data. BWS was developed in the late 1980s and first proposed in the early 1990s as an alternative to other preference elicitation approaches, such as discrete choice experiments [33]. In contrast to these, BWS requires respondents to identify both the best and the worst options in a choice set. Thus, respondents define the extremes of a latent, subjective continuum in which they are asked to choose the pair that maximize the value difference between the best and the worst item. This type of experiment is generally considered as a simpler task for respondents [34,35].

In our object case BWS, respondents are asked to select from each choice set one factor that had the most important influence on their decision (best) and one factor that had the least important influence on their decision (worst) whether to have primary genetic testing. We followed good practices to select the items and construct the choice sets [34–36]. This means that the selection of items was driven by the results of a scoping review (see supporting information S1 File including the search strategy as well as the number of identified and screened studies). Items identified were clustered into key topics related to genetic testing, including personal factors, testing structure/context, testing processes, outcomes (benefits vs. risks), and testing specifications. Based on the results and items extracted, we then developed an initial assessment of potential items, followed by a discussion within the research team (both clinical experts in the field of genetic testing as well as stated preferences researchers) to ensure that the most important items for the BWS were retained. The final list included 13 items covering primary genetic testing aspects like personal factors, testing processes and outcomes (benefits vs. risks). Table 1 lists the final 13 items.

**Table 1:** List of items for the BWS (primary genetic testing), derived from a scoping review and refined through expert discussions.

Aspect	Item
Personal factors	Someone in my family was diagnosed with the disease Someone in my family had genetic testing before
Processes	The genetic test includes an out-of-pocket fee that I must pay The time it takes for me to get results back from the genetic test
Outcomes (benefits vs. risks)	The genetic test results will support my own future healthcare decisions The genetic test results will inform my family members of their risk for the disease The genetic test results will help me with family planning I will need additional appointment(s) with my healthcare team and/or specialists if the genetic test results are positive The genetic test results will indicate what treatment will be necessary for disease management The genetic test results will provide information about disease prevention (like medical intervention, lifestyle change, screening) The genetic test results will provide information about risk of developing the disease and impact on life expectancy Potential that someone outside of my healthcare team could access my genetic test results The potential of getting an inaccurate genetic test result

The choice sets of the BWS were designed using a balanced incomplete block design (BIBD). In such a design, each item appears an equal number of times across all choice sets (i.e., combination of items) and each pair of items appears together an equal number of times ensuring a balance across different blocks [37,38]. BIBD designs are characterized by (1) orthogonality (items are shown and paired an approximately equal number of times), (2) minimal overlap (minimizing the number of times each item appears within the same choice set across the design), and (3) balance (items appear approximately an equal number of times in each position) [39]. In total, 13 choice sets with four items per choice set will be used to be able to estimate both main effects and possible selected interaction effects. We avoided blocking as our sample is relatively small. The software RStudio Version 09.1 was used to construct the experimental design (see supporting information S2 File).

Additional to the BWS for the primary genetic testing, a ranking exercise to elicit preferences around secondary analyses will be included. Respondents are asked to order all items from most to least important based on their preferences. Unlike BWS, where items are assessed in subsets, ranking ensures a complete and direct prioritization of all items at once, which is suitable as there is only a small number of items influencing decision-making for secondary analyses. The approach for identifying items for the ranking exercise (secondary analyses) followed the same procedure as for the BWS (primary genetic testing). Based on the results of the scoping review (see supplementary information S1 File), the six most important items for secondary analyses were selected (see Table 2). Furthermore, respondents will have the chance to provide other items that impacted their decision-making as a free-text answer.

**Table 2:** List of items for the ranking exercise (secondary analyses), derived from a scoping review and refined through expert discussions.

Obtaining information about another disease that I can act upon
Obtaining information to inform my relatives about another disease
Experiencing distress or anxiety in case of receiving a positive result
Experiencing uncertainty of not knowing about my risk for another disease
Receiving support from, or being fully informed by genetics healthcare professionals about the impacts of getting information about potentially other diseases
Deciding who has access to the results from the secondary analysis

### 2.2. Survey format

An online, self-administered, cross-sectional survey was created using Qualtrics software that contains three sections. The online survey is compatible with both web and mobile versions and scales the questions automatically depending on the detected device type (phone, tablet and laptop). Section one: introduction, background, consent, sociodemographic data (e.g., gender, age, education, ethnicity, household income), health status, comorbidities, and health literacy. Section two: BWS for primary genetic testing, ranking exercise for secondary analyses and a willingness to pay task for out-of-pocket costs for genetic testing in general. Section three: experience with genetic testing (e.g., knowledge, attitudes, expectations). The full survey instrument can be found in supporting information (S3 File).

To reduce hypothetical biases and to engage respondents in providing thoughtful answers, only one choice set per screen will be presented, with less scrolling as possible. Prior to answering the first BWS task, respondents are provided instructions and an example BWS choice set. The survey draft was initially reviewed and pre-tested internally by members of the research team. It was then pre-tested prior to fielding using a think-aloud approach to evaluate comprehension, survey flow and task difficulty [40,41]. Changes made included editorial changes, minor changes to improve clarity, simplification and reordering of some questions, and formatting and layout changes to enhance user experiences on both web and mobile versions. Within the survey instrument, there are no back buttons to force respondents in answering all questions before being able to proceed with the survey. The whole survey will take approximately 15–20 minutes to complete.

### 2.3. Study population and recruitment

The main target population are individuals at moderate risk of breast cancer or aortic disease enrolled in the PreventGene study. Breast cancer and aortic disease were chosen as they are frequent reasons for clinical genetics referrals in Alberta based on our study team members’ experiences. They also represent two prominent sub-specialties in clinical genetics (cancer genetics and cardiac genetics) [42,43]. The inclusion and exclusion criteria for breast cancer were based on the Alberta Health Services Hereditary Cancer Clinic’s (HCC) genetic testing guidelines (see supporting information S4 File), which aligns with the National Comprehensive Cancer Network guidelines [44]. As clinical genetic testing is indicated for high-risk individuals only, potential participants with personal and/or family history of breast cancer who do not satisfy the HCC guidelines are considered at moderate risk and therefore eligible for study enrolment. For aortic disease, the inclusion criteria were informed through discussions among clinical experts in the research team, as no formal screening criteria are available for aortic disease in Alberta, Canada. In total, the study will include n = 350 individuals from three different cohorts:

1. Cohort 1 (n = 150): Female individuals with a personal history of breast cancer (diagnosed ≤65 years old) who do not meet the provincial criteria in Alberta, Canada, for clinical hereditary breast cancer testing; OR female individuals (>20 years old) with no personal history of breast cancer with one first degree relative with breast cancer; OR female individuals (>20 years old) with no personal history of breast cancer with two affected relatives of any degree (diagnosed ≤65 years old);
2. Cohort 2 (n = 150): Non-syndromic male and female individuals (>50 years old) with aortic aneurysm and no relatives (<55 years old) with aortic aneurysm; and
3. Cohort 3 (n = 50): Individuals who meet the criteria to participate in cohorts 1 or 2 and chosen not to have any genetic testing but agreed to participate in the survey.

These three different cohorts will enable us to estimate and understand differences in preferences of those who chose not to have genetic testing (cohort 3) compared to those who chose to have genetic testing (cohorts 1 and 2) (study aim 2). Determining the appropriate sample size for BWS studies is a challenge as there are no established guidelines currently available [39]. However, the minimum required sample size can vary between several factors, such as question complexity, blocking, and necessity for subgroup analyses [45]. Existing stated preference studies typically involve 100 and 300 respondents [46]. Therefore, we chose a sample size of n = 350 individuals for this study.

All individuals will be recruited through an initial genetic counselling consultation by the genetics counsellor (LT) via referrals by their healthcare providers to the Genetics Cancer Program or Breast Surgical/Oncology (cohort 1) or by the attending geneticist at the Genetic Connective Tissue clinic (cohort 2). Within one week of making the decision for or against having genetic testing for primary testing and/or secondary analyses, the respondents will be asked to fill in the survey via the sent email invitations. Each cohort will receive the same survey. To ensure that participants cannot submit duplicate responses, the email invitations include a study identification and the participants’ provided email address, which are linked to the survey to prevent multiple submissions. To incentivize individuals who decided against testing (cohort 3) to participate in the survey, they will be given the option to enter a gift card lottery (5x$50) and will be eligible for the lottery after fully completing the survey. One general reminder will be sent out to individuals who have not started the survey one week after survey invitation and on an individual basis in case of incomplete surveys. Participant recruitment at the genetics clinics started on February 18, 2025, and data collection started on March 2, 2025. Both recruitment and data collection are ongoing and are expected to be finished by spring 2026.

### 2.4. Ethics statement

The study is approved by the Conjoint Health Research Ethics Board of the University of Calgary (ethics ID: REB23–1663). At the start of the survey, a page containing the consent form and further details of the study is presented (e.g., purpose, duration, benefits, risks). Written online informed consent for the sub-study will be obtained from all participants, with implied consent to participate in the study when they proceed with the survey beyond the consent form page. Participants are free to stop their voluntary participation in the study at any time by closing the survey. The response up to that point will be recorded. In addition, for cohort 3 only, the details of the lottery are described. Furthermore, contact information of the research team will be provided for any questions that may arise.

Participants’ email addresses will only be used for the purpose of sending unique, single-use survey links, gift cards (cohort 3 only, if they wish to enter the gift card lottery), and reminders to complete the survey (if necessary); their emails will be destroyed after data collection is complete. All survey data downloaded from Qualtrics will be password-protected and securely stored on the University of Calgary’s OneDrive for five years after the study ends. Any future use of the collected data will be required to undergo a review by the Conjoint Health Research Ethics Board of the University of Calgary.

## 3. Analytical plan

The planned analyses must take into account the panel structure of the BWS data with the same respondent providing multiple outcomes (best and worst) for a sequence of different choice sets [37]. First, the data will be exported from Qualtrics into Microsoft Excel, and imported into Stata SE18 [47]. Respondent characteristics will be checked to determine the representativeness of the sample compared with the Canadian population living in the province of Alberta. Afterwards, statistical analyses will be carried out on the BWS responses to quantify the relative importance of the 13 items using a Maximum Difference Scaling (MaxDiff) model [39]. The initial analysis will start with a count analysis calculating the frequency that each item is selected as most and least important. The difference between the total of “most important” and “least important” responses will be calculated for each item (i.e., BWS score), which provides an indication of the relative importance of each item relative to the others. A ranking will be generated based on the normalized BWS scores, where higher positive scores indicate greater importance of an attribute [35,39]. We will then use multinomial logit model to estimate benefit-risk trade-offs, and we will examine preferences by subgroups (i.e., differences between cohorts 1 and 2). To further explore preference heterogeneity, we will apply both mixed logit models and latent class analysis. Mixed logit models allow for continuous variation in preferences across individuals, while latent class analysis identifies patterns of similar preferences based on respondents’ characteristics, including but not limited to sociodemographic factors, attitudes, and experiences, and estimates the probability that each respondent belongs to each class. If different preference patterns are observed, we will evaluate the association between participant characteristics and latent class membership [45].

## 4. Expected results and dissemination

This sub-study, as part of the PreventGene study, focuses on eliciting preferences (relative importance of factors that influence decision-making) of individuals at moderate risk of breast cancer or aortic disease for primary genetic testing and secondary analyses after they made their decision but before receiving their test results. To our knowledge, preference studies have so far focused on general populations with unknown risk factors [27,28] or high-risk individuals, and eliciting preferences before making the decision to get tested or after receiving the test results [24–26]. Only one study, to date, investigated high-risk individuals’ preferences for genetic testing of colorectal cancer risk after deciding to get tested, but before results disclosure [48]. Focusing on characteristics that influenced moderate-risk individuals’ decision-making for or against having genetic testing will help to better understand their decision-making process about what testing types (primary genetic testing and/or secondary analyses) should be available in which contexts (breast cancer and/or aortic disease) and for which individuals.

When designing and evaluating genetic testing programs for individuals at moderate risk whom genetic testing is not usually offered, it is important to understand the individual preferences on how they value various factors, which factors are perceived as the most and least important, and variations between subgroups to optimize patient-centered care [49]. First results will be expected by autumn 2026. The expected results can inform clinical, policy and regulatory decision makers in planning, offering, and reimbursing additional future genetic testing programs for individuals at moderate risk.

This study may have potential limitations: Firstly, it might be difficult to collect data during the clinical process, which can lead to non-response bias and low sample size. We will try to reduce non-responses by monitoring the data collection process continuously and sending out reminders, if needed. Secondly, there may occur a recall bias as we will ask individuals about the most and least important factors for or against genetic testing after they already made the decision. We will try to minimize recall bias as we intend to send out the survey instrument immediately after the genetic consultation and ask participants to complete the survey within one week, if possible. However, we do not know if that was different from prior to the decision and if that is different from after receiving results. Thirdly, as the items used in the BWS cover only general aspects of genetic testing, the findings of this study may not be generalizable to other genetic testing procedures (e.g., prenatal testing or carrier screening) or disease areas (e.g., rare diseases). Furthermore, additional aspects of genetic testing could be relevant, however, the systematic development process and pre-testing suggest that the most important drivers of decision-making were captured. Lastly, this study aims to understand preferences for genetic testing in individuals at moderate risk only. However, considering shared decision-making, future studies may consider including the clinician preferences as well.

Data dissemination will occur through academic publications in high-ranked peer-reviewed journals and during academic conferences after data analyses is finalized (expected autumn/winter 2026). Further changes to this study will be available online on the Open Science Framework (10.17605/OSF.IO/JFPH9).

## Supporting information

S1 File Results of the scoping review. (PDF)

S2 File Experimental design for the BWS. (PDF)

S3 File Survey instrument implemented in Qualtrics. (PDF)

S4 File Clinical genetic testing eligibility guidelines for breast cancer. (PDF)
