# Supplementary material for: To test or not to test? Study protocol for a best-worst scaling to understand decision-making and preferences for genetic testing in moderate-risk individuals
Source: PLoS One. 2025 Dec 29;20(12):e0339696. doi: 10.1371/journal.pone.0339696 (PMC12747399; doi:10.1371/journal.pone.0339696)
Supplement: S1 File — (PDF) [file pone.0339696.s001.pdf]

Supporting information: PLOS One

To test or not to test? Study protocol for a best-worst scaling to understand decision-making and preferences for genetic testing in moderate-risk individuals

Carina Oedingen[1], Nicolle Hua[1], Karen V. MacDonald[1], Julien Marcadier[2,3], Renee Perrier[2,3], Lindsay Tuer[2], Brenda McInnes[2,3], Francois Bernier[2,3], Deborah A. Marshall[1,3]

1 Department of Community Health Sciences, Cumming School of Medicine, University of Calgary, Calgary, Alberta, Canada

2 Department of Medical Genetics, Cumming School of Medicine, University of Calgary, Calgary, Alberta, Canada

3 Alberta Children's Hospital Research Institute, Calgary, Alberta, Canada

Supporting information: PLOS One

To test or not to test? Study protocol for a best-worst scaling to understand decision-making and preferences for genetic testing in moderate-risk individuals

S1. Results of the scoping review

Search strategy in PubMed based on PICO framework

#	Query	Results (2024-09-04)
1	"gene* test*"[Title/Abstract] OR "gene* profil*"[Title/Abstract] OR "gene* panel"[Title/Abstract] OR "gene* * test*"[Title/Abstract] OR "gene* sequencing*"[Title/Abstract] OR "gene* * sequencing*"[Title/Abstract] OR "gene* * profil*"[Title/Abstract] OR "gene* * panel*"[Title/Abstract]	202,346
2	"DNA test*"[Title/Abstract] OR "DNA * test*"[Title/Abstract] OR "DNA sequencing"[Title/Abstract] OR "DNA * sequencing*"[Title/Abstract]	41,609
3	"genom* test*"[Title/Abstract] OR "genom* * test*"[Title/Abstract] OR "genom* sequencing*"[Title/Abstract] OR "genom* * sequencing*"[Title/Abstract]	55,708
4	"genome sequencing"[Title/Abstract] OR "whole genome sequencing"[Title/Abstract] OR "whole exome sequencing"[Title/Abstract] OR "exome sequencing"[Title/Abstract]	73,293
5	"pharmacogen*"[Title/Abstract] OR "pharmacogen* test*"[Title/Abstract] OR "pharmacogen* * test*"[Title/Abstract]	22,563
6	"precision medicine"[Title/Abstract]	25,195
7	"personali*ed medicine"[Title/Abstract] OR "personali*ed * medicine"[Title/Abstract]	24,151
8	"next-generation sequencing"[Title/Abstract] OR "next generation sequencing"[Title/Abstract] OR "next-generation technolog*"[Title/Abstract] OR "next generation technolog*"[Title/Abstract]	64,967
9	"predispositional * test*"[Title/Abstract] OR "predictive test*"[Title/Abstract] OR "predictive * test*"[Title/Abstract] OR "presymptomatic test*"[Title/Abstract] OR "presymptomatic * test*"[Title/Abstract] OR "pre-symptomatic test*"[Title/Abstract] OR "pre-symptomatic * test*"[Title/Abstract]	3,755

To test or not to test? Study protocol for a best-worst scaling to understand decision-making and preferences for genetic testing in moderate-risk individuals

10	#1 OR #2 OR #3 OR #4 OR #5 OR #6 OR #7 OR #8 OR #9	371,282
11	"stated prefer*"[Title/Abstract]	1,135
12	"patient prefer*"[Title/Abstract]	13,559
13	“patient cho*"[Title/Abstract]	2,818
14	“choice behavior*”[Title/Abstract]	1,869
15	"patient decision*"[Title/Abstract]	3,004
16	“uncertainty”[Title/Abstract]	112,600
17	"benefit-risk*"[Title/Abstract] OR "benefit* risk*"[Title/Abstract] OR "risk-benefit*"[Title/Abstract] OR "risk* benefit*"[Title/Abstract]	20,468
18	"trade-off*”[Title/Abstract]	36,107
19	“preference*”[Title/Abstract]	208,931
20	#11 OR #12 OR #13 OR #14 OR #15 OR #16 OR #17 OR #18 OR #19	376,942
21	"patient*"[Title/Abstract] OR “parent*”[Title/Abstract]	9,059,970
22	"public"[Title/Abstract]	701,545
23	#21 OR #22	9,580,894
24	#10 AND #20 AND #23	2,378
25	#24 Filter: **In the last 5 years**	1,121

Supporting information: PLOS One

To test or not to test? Study protocol for a best-worst scaling to understand decision-making and preferences for genetic testing in moderate-risk individuals

Flowchart of identified studies

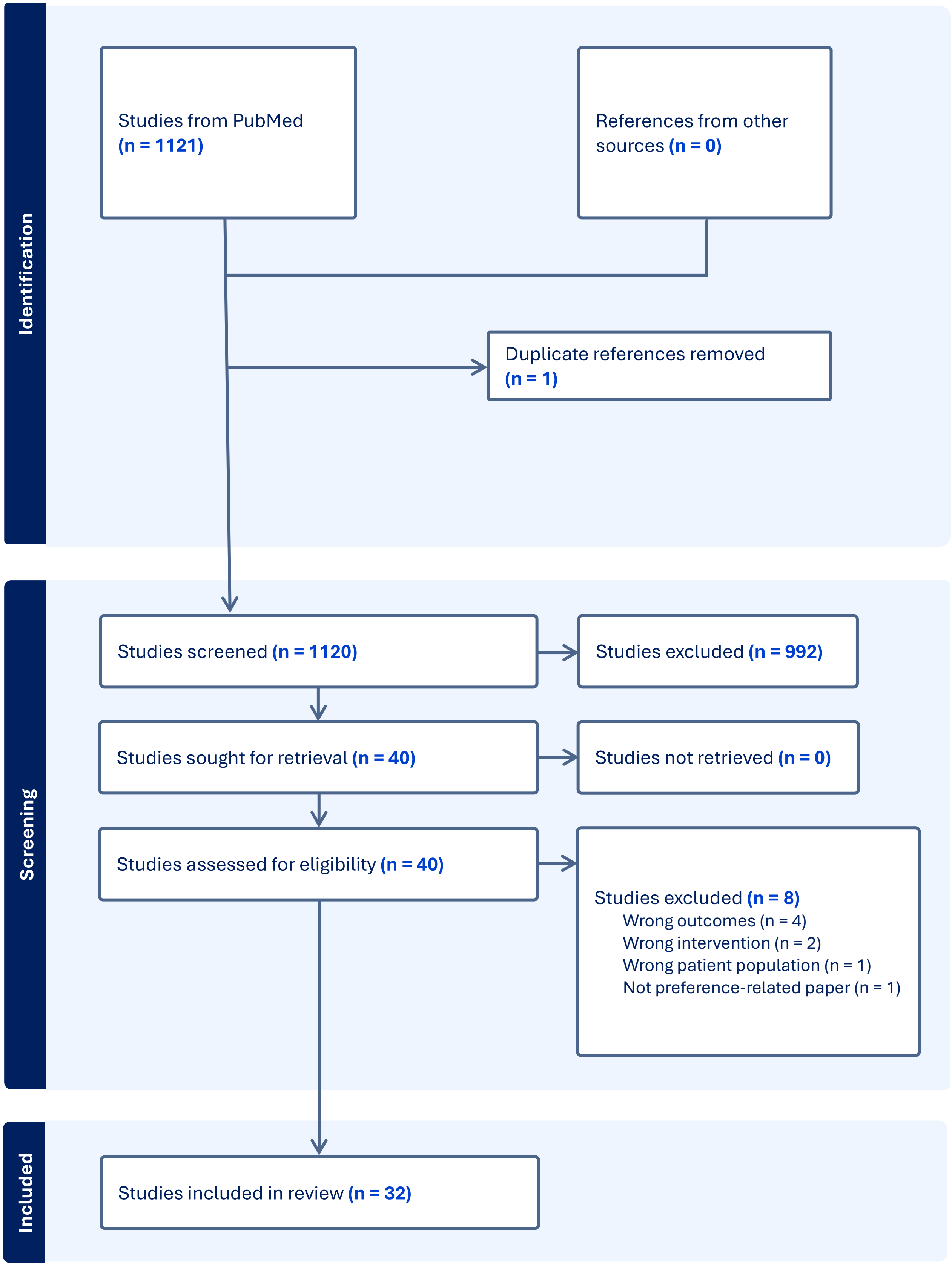

Supporting information: PLOS One

To test or not to test? Study protocol for a best-worst scaling to understand decision-making and preferences for genetic testing in moderate-risk individuals

Extracted items from the scoping review and clustered based on key aspects around genetic testing

Personal factors	Structure/ Context	Processes	Outcomes (benefits/risks)	Testing
Disease severity	*Testing location*	*Cost of testing (Out-of-pocket)*	*Identify best medication or dose / Drug effectiveness / Predict initial dosing / Optimize current medications*	*Number of disease or genes tested for*
Family history	*Testing availability or eligibility*	*Distance to appointment*	*Identify or prevent potential side effects / Predict serious side effects / Reduce medication burden / Predict mild side effects*	*Disease or gene type*
Gene predisposition	*Testing preparations (sample type)*	*Duration of appointment*	*Treatment availability for disease covered by the test*	*Prevision of tests or type of test*
Probability of having disease / Disease worries	*Physicians' recommendation*	*Number of appointments required / follow-up appointments needed*	*Support future healthcare decisions / Optimize future healthcare decisions*	*Information about risk of development / Disease development / Disease prediction*
Age of onset of disease	*Data availability, storage, and privacy*	*Waiting or turnaround time for genetic results*	*Impact of results on families / Impact of results on family planning*	*Risk of disease severity*
Peer or herd behavior		*Communication of test results / Type of healthcare provider*	*Life insurance impacts*	*Test-related risks (sensitivity, specificity) / Test accuracy*
Motivations around genetic testing			*Prevention, treatment, or lifestyle changes / Impact on quality of life*	*Test results may contain information about unrelated health risks*

To test or not to test? Study protocol for a best-worst scaling to understand decision-making and preferences for genetic testing in moderate-risk individuals

Involvement or advocacy of own treatment decisions			*Screening impact*	*Incidental findings*
			Survival / Mortality	
			Impact on work opportunities	
			Risk of misinterpretation and take inappropriate actions	
			Psychological distress (receiving results)	
			Risk that someone outside of healthcare team could access genetic results	
			Early detection	
