# Supplementary material for: To test or not to test? Study protocol for a best-worst scaling to understand decision-making and preferences for genetic testing in moderate-risk individuals
Source: PLoS One. 2025 Dec 29;20(12):e0339696. doi: 10.1371/journal.pone.0339696 (PMC12747399; doi:10.1371/journal.pone.0339696)
Supplement: S2 File — (PDF) [file pone.0339696.s002.pdf]

Supporting information: PLOS One

To test or not to test? Study protocol for a best-worst scaling to understand decision-making and preferences for genetic testing in moderate-risk individuals

Carina Oedingen[1], Nicolle Hua[1], Karen V. MacDonald[1], Julien Marcadier[2,3], Renee Perrier[2,3], Lindsay Tuer[2], Brenda McInnes[2,3], Francois Bernier[2,3], Deborah A. Marshall[1,3]

1 Department of Community Health Sciences, Cumming School of Medicine, University of Calgary, Calgary, Alberta, Canada

2 Department of Medical Genetics, Cumming School of Medicine, University of Calgary, Calgary, Alberta, Canada

3 Alberta Children's Hospital Research Institute, Calgary, Alberta, Canada

Supporting information: PLOS One

To test or not to test? Study protocol for a best-worst scaling to understand decision-making and preferences for genetic testing in moderate-risk individuals

S2. Experimental design for the BWS

Design

Choice task #	Row 1	Row 2	Row 3	Row 4
1	1	2	5	7
2	2	4	10	12
3	4	5	6	11
4	1	8	10	11
5	3	5	9	10
6	6	7	10	13
7	1	4	9	13
8	3	4	7	8
9	5	8	12	13
10	2	6	8	9
11	7	9	11	12
12	2	3	11	13
13	1	3	6	12

Item balance (rho; 1st order balance)

Item	# of times item occurs across tasks
1	4
2	4
3	4
4	4
5	4
6	4
7	4
8	4
9	4
10	4
11	4

12	4
13	4

Item Co-balance (lambda; 2nd order balance)

Item	# of times each item co-occurs in a task throughout the design											
	**1**	**2**	**3**	**4**	**5**	**6**	**7**	**8**	**9**	**10**	**11**	**12**	**13**
**1**	0	1	1	1	1	1	1	1	1	1	1	1	1
**2**	1	0	1	1	1	1	1	1	1	1	1	1	1
**3**	1	1	0	1	1	1	1	1	1	1	1	1	1
**4**	1	1	1	0	1	1	1	1	1	1	1	1	1
**5**	1	1	1	1	0	1	1	1	1	1	1	1	1
**6**	1	1	1	1	1	0	1	1	1	1	1	1	1
**7**	1	1	1	1	1	1	0	1	1	1	1	1	1
**8**	1	1	1	1	1	1	1	0	1	1	1	1	1
**9**	1	1	1	1	1	1	1	1	0	1	1	1	1
**10**	1	1	1	1	1	1	1	1	1	0	1	1	1
**11**	1	1	1	1	1	1	1	1	1	1	0	1	1
**12**	1	1	1	1	1	1	1	1	1	1	1	0	1
**13**	1	1	1	1	1	1	1	1	1	1	1	1	0
