# Supplementary material for: To test or not to test? Study protocol for a best-worst scaling to understand decision-making and preferences for genetic testing in moderate-risk individuals
Source: PLoS One. 2025 Dec 29;20(12):e0339696. doi: 10.1371/journal.pone.0339696 (PMC12747399; doi:10.1371/journal.pone.0339696)
Supplement: S3 File — (PDF) [file pone.0339696.s003.pdf]

Supporting information: PLOS One

To test or not to test? Study protocol for a best-worst scaling to understand decision-making and preferences for genetic testing in moderate-risk individuals

Carina Oedingen[1], Nicolle Hua[1], Karen V. MacDonald[1], Julien Marcadier[2,3], Renee Perrier[2,3], Lindsay Tuer[2], Brenda McInnes[2,3], Francois Bernier[2,3], Deborah A. Marshall[1,3]

1 Department of Community Health Sciences, Cumming School of Medicine, University of Calgary, Calgary, Alberta, Canada

2 Department of Medical Genetics, Cumming School of Medicine, University of Calgary, Calgary, Alberta, Canada

3 Alberta Children's Hospital Research Institute, Calgary, Alberta, Canada

Supporting information: PLOS One

To test or not to test? Study protocol for a best-worst scaling to understand decision-making and preferences for genetic testing in moderate-risk individuals

S3. Survey instrument implemented in Qualtrics

[This survey is a web-based survey. Therefore, the display is different compared to the implementation into Qualtrics]

Survey on Preferences for Genetic Testing

Welcome!

The University of Calgary in collaboration with Alberta Health Services are conducting a study about your preferences for genetic testing. The survey will take about 15-20 minutes to complete.

If you are completing the survey on a mobile device like a smartphone, we recommend rotating your phone sideways (landscape mode) when answering certain questions for the best experience.

SPONSOR: Calgary Health Foundation

INVESTIGATORS:

**University of Calgary, Department of Medical Genetics**	**Dr. Francois Bernier,** Professor *(Principal Investigator)*
**Richmond Road Diagnostic and Treatment Centre**	**Dr. Julien Marcadier**, Medical Geneticist<br>**Dr. Renee Perrier**, Medical Geneticist
**University of Calgary, Department of Community Health Sciences**	**Dr. Deborah Marshall**, Professor<br>**Dr. Carina Oedingen,** Postdoctoral Research Fellow<br>**Karen MacDonald,** Research Scientist, Research Manager

--- Page Break (Qualtrics) ---

Authentication *[Participants will input their email addresses and study IDs – both are provided in an automated email by the research coordinator LT]*

Note: Inputted study ID will determine which consent form is shown to the participant (1000 – 2999 = cohort 1 + 2 consent; 3000 – 3999 = cohort 3 consent)

To test or not to test? Study protocol for a best-worst scaling to understand decision-making and preferences for genetic testing in moderate-risk individuals

--- Page Break (Qualtrics) ---

Implied Consent to Participate in Research

[For cohort 1 and 2]

INTRODUCTION

Dr. Francois Bernier and associates from the Cumming School of Medicine at the University of Calgary are conducting a research study.

This consent form is only part of the process of informed consent. It should give you the basic idea of what the research is about and what your participation will involve. If you would like to get more detail about something mentioned here, or information not included here, please ask. Take the time to read this carefully and to understand any accompanying information.

You are invited to be in this study because you have a family or personal history of breast cancer OR aortic disease, and you are NOT eligible for genetic testing through routine medical care. Your participation in this research study is voluntary.

WHY IS THIS STUDY BEING DONE?

We are conducting a survey to understand preferences related to moderate risk genetic testing. Information from the survey will help with the planning and development of future genetic health programs. This survey is part of the PreventGene (Precision Medicine Expansion to Moderate Risk Patients) study to determine the benefits of offering genetic testing to people who are classified as moderately likely to have a disease-causing variant (moderate risk).

HOW MANY PEOPLE WILL TAKE PART IN THIS STUDY?

About 350 people will take part in this study Alberta wide through the University of Calgary.

WHAT WILL HAPPEN IF I TAKE PART IN THIS RESEARCH STUDY?

Participation in this study involves completing a one-time online survey. In the survey, you will be asked questions about your experiences with genetic testing, your preferences for genetic testing, and what impacted your decision to have (or not have) genetic testing. The survey will take about 15-20 minutes to complete.

ARE THERE ANY POTENTIAL RISKS OR DISCOMFORTS THAT I CAN EXPECT FROM THIS STUDY?

There are no foreseeable risks from completing the survey.

HOW LONG WILL I BE IN THIS STUDY?

Participation in this study involves completing a one-time online survey. The survey will take about 15-20 minutes to complete.

ARE THERE ANY POTENTIAL BENEFITS IF I PARTICIPATE?

There will be no direct benefit to you from participating in this study. However, this study will help with the planning and development of future genetic health programs.

CAN I STOP BEING IN THE STUDY?

Participation in this study is voluntary and you can decide to stop the survey at any time by closing the browser and exiting the survey. Your responses up to the point you stop will be recorded. You may request to have your survey responses withdrawn by contacting the study coordinator. Once the results are analyzed, it will no longer be possible to withdraw from the study.

WILL INFORMATION ABOUT ME AND MY PARTICIPATION BE KEPT CONFIDENTIAL?

The information collected will only be used for this study and no identifiable information about you will be kept with the research data. All electronic study data will be stored on secured computer servers at the University of Calgary. Qualtrics is an online survey platform with servers located in Toronto, Ontario, Canada. All survey data are encrypted and stored directly on its servers. Researcher access to the survey data is password-protected and the transmission is encrypted.

HOW LONG WILL INFORMATION FROM THE STUDY BE KEPT?

The researchers intend to keep the research data for 5 years after the study ends as per University of Calgary protocol. Any future use of this research data is required to undergo review by a Research Ethics Board.

--- Page Break (Qualtrics) ---

Implied Consent to Participate in Research

[For cohort 3]

INTRODUCTION

Dr. Francois Bernier and associates from the Cumming School of Medicine at the University of Calgary are conducting a research study.

This consent form is only part of the process of informed consent. It should give you the basic idea of what the research is about and what your participation will involve. If you would like to get more detail about something mentioned here, or information not included here, please ask. Take the time to read this carefully and to understand any accompanying information.

You are invited to be in this study because you have a family or personal history of breast cancer OR aortic disease, and you are NOT eligible for genetic testing through routine medical care. Your participation in this research study is voluntary.

WHY IS THIS STUDY BEING DONE?

We are conducting a survey to understand preferences related to moderate risk genetic testing. Information from the survey will help with the planning and development of future genetic health programs. This survey is part of the PreventGene (Precision Medicine Expansion to Moderate Risk Patients) study to determine the benefits of offering genetic testing to people who are classified as moderately likely to have a disease-causing variant (moderate risk).

To test or not to test? Study protocol for a best-worst scaling to understand decision-making and preferences for genetic testing in moderate-risk individuals

HOW MANY PEOPLE WILL TAKE PART IN THIS STUDY?

About 350 people will take part in this study Alberta wide through the University of Calgary.

WHAT WILL HAPPEN IF I TAKE PART IN THIS RESEARCH STUDY?

Participation in this study involves completing a one-time online survey. In the survey, you will be asked questions about your experiences with genetic testing, your preferences for genetic testing, and what impacted your decision to have (or not have) genetic testing. The survey will take about 15-20 minutes to complete.

ARE THERE ANY POTENTIAL RISKS OR DISCOMFORTS THAT I CAN EXPECT FROM THIS STUDY?

There are no foreseeable risks from completing the survey.

HOW LONG WILL I BE IN THIS STUDY?

Participation in this study involves completing a one-time online survey. The survey will take about 15-20 minutes to complete.

ARE THERE ANY POTENTIAL BENEFITS IF I PARTICIPATE?

There will be no direct benefit to you from participating in this study. However, this study will help with the planning and development of future genetic health programs.

CAN I STOP BEING IN THE STUDY?

Participation in this study is voluntary and you can decide to stop the survey at any time by closing the browser and exiting the survey. Your responses up to the point you stop will be recorded. You may request to have your survey responses withdrawn by contacting the study coordinator. Once the results are analyzed, it will no longer be possible to withdraw from the study.

WILL I BE PAID FOR PARTICIPATING, OR DO I HAVE TO PAY FOR ANYTHING

You will not have to pay for participating in this study. You will not be paid for participating in this study. However, there will be given the opportunity to enter a lottery for the chance to win a $50 gift card. Up to five (5) winners will be selected (one gift card per winner only). Participants who wish to be entered into the lottery must provide a valid email address.

WILL INFORMATION ABOUT ME AND MY PARTICIPATION BE KEPT CONFIDENTIAL?

The information collected will only be used for this study and no identifiable information about you will be kept with the research data. All electronic study data will be stored on secured computer servers at the University of Calgary. Qualtrics is an online survey platform with servers located in Toronto, Ontario, Canada. All survey data are encrypted and stored directly on its servers. Researcher access to the survey data is password-protected and the transmission is encrypted.

HOW LONG WILL INFORMATION FROM THE STUDY BE KEPT?

The researchers intend to keep the research data for 5 years after the study ends as per University of Calgary protocol. Any future use of this research data is required to undergo review by a Research Ethics Board.

--- Page Break (Qualtrics) ---

Research Study Contacts

The Research Team:

You may contact Dr. Carina Oedingen or Karen MacDonald preventgenesurvey@ucalgary.ca with any questions or concerns about the research or your participation in this study.

Conjoint Health Research Ethics Board (CHREB):

If you have any questions concerning your rights as a possible participant in this research, please contact the Chair, Conjoint Health Research Ethics Board, University of Calgary at 403-220-7990 or email chreb@ucalgary.ca.

--- Page Break (Qualtrics) ---

Agreement to Participate

Your decision to complete the survey will be interpreted as an indication of your agreement to participate. In no way does this waive your legal rights nor release the investigators or involved institutions from their legal and professional responsibilities. You are free to withdraw from the study at any time.

--- Page Break (Qualtrics) ---

Lottery Participation *[cohort 3 only]*

Would you like to enter the lottery for a chance to win one of five $50 gift cards? If you win, we will contact you using the same email address you provided to participate in this study.

If you select "Yes", please make sure to complete this survey to be eligible for the lottery.

- ○ Yes, I wish to enter the lottery
- ○ No, I do not wish to enter the lottery

--- Page Break (Qualtrics) ---

To test or not to test? Study protocol for a best-worst scaling to understand decision-making and preferences for genetic testing in moderate-risk individuals

General Questions About You

We would first like to ask you some questions about your personal situation.

1. *Which gender do you identify as?*
   - Man
   - Woman
   - Other (please specify): [free text answer]
   - Prefer not to answer

2. *How old are you (in years)?*
   [free text answer *"Enter a number here"]*]

--- Page Break (Qualtrics) ---

3. *What is the highest level of education you have completed?*
   - Some high school
   - High school diploma
   - Diploma from trade school or college
   - Some university
   - University degree
   - Other (please specify): [free text answer]

4. *What is your current employment status?*
   - Employed
     (full time, part time, self-employment, seasonal employment)
   - Employed but on leave
     (maternity/parental leave, disability leave/long term disability, medical leave)
   - Unemployed
     (students, retirement, home makers)

5. *What is your household income?*
   - Less than $20,000
   - $20,000-$39,999
   - $40,000-$59,999
   - $60,000-$79,999
   - $80,000-$99,999
   - $100,000 or more
   - Prefer not to answer

--- Page Break (Qualtrics) ---

6. *Do you have children (any age)?*
   - Yes, I have children
   - No, I do not have children

7. *You may belong to one or more population groups in the list below. Please select the one that applies. Select 'multiple ethnicities' if you belong to more than one of the options listed or select 'other' if you belong to one not listed here.*
   - Indigenous peoples
   - Caucasian
   - South Asian
   - Chinese
   - African Canadian
   - Filipino
   - Latin American
   - Arab
   - Southeast Asian
   - West Asian
   - Korean
   - Japanese
   - Multiple ethnicity
   - Other ethnicity not listed here
   - Unknown
   - Prefer not to answer

--- Page Break (Qualtrics) ---

7a. [Filter question for those indicated choice "n" in Q7] You identified with another ethnicity that was not previously listed. With which population group do you identify? Please specify using free text below.

[free text answer]

8. *Are you religious?*
   - Yes
   - No
   - Prefer not to answer

9. *Do you have private or employer paid health insurance (for example, a group benefits plan)?*
   - Yes
   - No

- I am not sure

--- Page Break (Qualtrics) ---

10. On a scale from strongly disagree to strongly agree, please indicate your level of agreement for each of the following statements:

	Strongly disagree	Moderately disagree	Slightly disagree	Slightly agree	Moderately agree	Strongly agree
I don't like situations that are uncertain	o	o	o	o	o	o
I feel uncomfortable when I don't understand the reason why an event occurred in my life	o	o	o	o	o	o
When I am confused about an important issue, I feel very upset	o	o	o	o	o	o
I'd rather know bad news than stay in a state of uncertainty	o	o	o	o	o	o

--- Page Break (Qualtrics) ---

11. How would you rate your health in general?
- Excellent
- Very good
- Good
- Fair
- Poor

12. Please indicate if you currently have one or more of the following medical problems. Select all that apply.

- ◻ Heart disease
- ◻ High blood pressure
- ◻ Lung disease
- ◻ Diabetes
- ◻ Ulcer or stomach disease
- ◻ Kidney disease
- ◻ Liver disease
- ◻ Anemia or other blood disease
- ◻ Cancer
- ◻ Depression
- ◻ Osteoarthritis/degenerative arthritis
- ◻ Back pain
- ◻ Rheumatoid arthritis
- ◻ Other medical problems (please specific) [free text answers]
- ◻ I do not currently have any medical problems [answer exclusive]

13. How concerned are you about developing severe health problems in the future?

- ○ Not at all worried
- ○ Slightly worried
- ○ Moderately worried
- ○ Very worried
- ○ Extremely worried

--- Page Break (Qualtrics) ---

14. On a scale from never to always, please indicate how you feel about each of the following statements:

	Never	Occasionally	Sometimes	Often	Always
How often do you have someone (like a family member, friend, hospital/ clinic worker or caregiver) help you read hospital materials?	○	○	○	○	○
How often do you have problems learning about your medical condition because of difficulty understanding written information?	○	○	○	○	○
How confident are you filling out medical forms by yourself?	○	○	○	○	○

--- Page Break (Qualtrics) ---

To test or not to test? Study protocol for a best-worst scaling to understand decision-making and preferences for genetic testing in moderate-risk individuals

Your Preferences for Genetic Testing

In this section of the survey, we would like to learn more about what impacted your decision to have (or not have) primary genetic testing and secondary analysis.

Primary genetic testing covers genes that are related to breast cancer or aortic diseases. **Secondary analysis** covers genes that are not related to breast cancer or aortic diseases but could provide information about your underlying risk for other diseases.

--- Page Break (Qualtrics) ---

Primary Genetic Testing

In the following questions, we will describe different aspects of genetic testing that can impact someone's decision to have (or not have) primary genetic testing, which includes genes that are related to breast cancer or aortic diseases. **There are no right or wrong answers. We are only interested in your opinion.** Let's start with an example.

****

Thinking back to the decision you made to have (or not have) primary genetic testing: What was the most important aspect and what was the least important aspect in your decision?

*Please select only one aspect in each column under "**Most important**" and "**Least important**".*

Most Important		Least Important
◉	Someone in my family was diagnosed with the disease	○
○	The genetic test includes an out-of-pocket fee that I must pay	◉
○	The genetic test results will inform my family members of their risk for the disease	○
○	Potential that someone outside of my healthcare team could access my genetic test results	○

The individual who answered the example question above felt that, out of the four items, having someone in their family diagnosed with the disease was the **most important** aspect and the out-of-pocket fee was the **least important** aspect in their decision-making when offered primary genetic testing. They made this choice by reading the list of items, then considering the impact that each one would have on their decision-making.

To test or not to test? Study protocol for a best-worst scaling to understand decision-making and preferences for genetic testing in moderate-risk individuals

****

The following pages have 13 questions like the example you just saw. The instructions are the same for each question.

If you are completing this section on a mobile device like a smartphone, we recommend rotating your phone sideways (landscape mode) for the best experience.

--- Page Break (Qualtrics) ---

Thinking back to the decision you made to have (or not have) primary genetic testing: What was the most important aspect and what was the least important aspect in your decision?

*Please select only one aspect in each column under "**Most important**" and "**Least important**".*

Question 1 of 13

**Most Important**		**Least Important**
	**(Item #1) Someone in my family was diagnosed with the disease**	
	**(Item #2) Someone in my family had genetic testing before**	
	**(Item #5) The genetic test results will support my own future healthcare decisions**	
	**(Item #7) The genetic test results will help me with family planning**	

--- Page Break (Qualtrics) ---

To test or not to test? Study protocol for a best-worst scaling to understand decision-making and preferences for genetic testing in moderate-risk individuals

Thinking back to the decision you made to have (or not have) primary genetic testing: What was the most important aspect and what was the least important aspect in your decision?

*Please select only one aspect in each column under "**Most important**" and "**Least important**".*

Question 2 of 13

**Most Important**		**Least Important**
	**(Item #2) Someone in my family had genetic testing before**	
	**(Item #4) The time it takes for me to get results back from the genetic test**	
	**(Item #10) The genetic test results will provide information about disease prevention (like medical intervention, lifestyle change, screening)**	
	**(Item #12) Potential that someone outside of my healthcare team could access my genetic test results**	

--- Page Break (Qualtrics) ---

Thinking back to the decision you made to have (or not have) primary genetic testing: What was the most important aspect and what was the least important aspect in your decision?

*Please select only one aspect in each column under "**Most important**" and "**Least important**".*

Question 3 of 13

**Most Important**		**Least Important**
	**(Item #4) The time it takes for me to get results back from the genetic test**	
	**(Item #5) The genetic test results will support my own future healthcare decisions**	
	**(Item #6) The genetic test results will inform my family members of their risk for the disease**	
	**(Item #11) The genetic test results will provide information about risk of developing the disease and impact on life expectancy**	

--- Page Break (Qualtrics) ---

To test or not to test? Study protocol for a best-worst scaling to understand decision-making and preferences for genetic testing in moderate-risk individuals

Thinking back to the decision you made to have (or not have) primary genetic testing: What was the most important aspect and what was the least important aspect in your decision?

*Please select only one aspect in each column under "**Most important**" and "**Least important**".*

Question 4 of 13

**Most Important**		**Least Important**
	**(Item #1) Someone in my family was diagnosed with the disease**	
	**(Item #8) I will need additional appointment(s) with my healthcare team and/or specialists if the genetic test results are positive**	
	**(Item #10) The genetic test results will provide information about disease prevention (like medical intervention, lifestyle change, screening)**	
	**(Item #11) The genetic test results will provide information about risk of developing the disease and impact on life expectancy**	

--- Page Break (Qualtrics) ---

Thinking back to the decision you made to have (or not have) primary genetic testing: What was the most important aspect and what was the least important aspect in your decision?

*Please select only one aspect in each column under "**Most important**" and "**Least important**".*

Question 5 of 13

**Most Important**		**Least Important**
	**(Item #3) The genetic test includes an out-of-pocket fee that I must pay**	
	**(Item #5) The genetic test results will support my own future healthcare decisions**	
	**(Item #9) The genetic test results will indicate what treatment will be necessary for disease management**	
	**(Item #10) The genetic test results will provide information about disease prevention (like medical intervention, lifestyle change, screening)**	

--- Page Break (Qualtrics) ---

To test or not to test? Study protocol for a best-worst scaling to understand decision-making and preferences for genetic testing in moderate-risk individuals

Thinking back to the decision you made to have (or not have) primary genetic testing: What was the most important aspect and what was the least important aspect in your decision?

*Please select only one aspect in each column under "**Most important**" and "**Least important**".*

Question 6 of 13

Most Important		Least Important
	**(Item #6) The genetic test results will inform my family members of their risk for the disease**	
	**(Item #7) The genetic test results will help me with family planning**	
	**(Item #10) The genetic test results will provide information about disease prevention (like medical intervention, lifestyle change, screening)**	
	**(Item #13) The potential of getting an inaccurate genetic test result**	

--- Page Break (Qualtrics) ---

Thinking back to the decision you made to have (or not have) primary genetic testing: What was the most important aspect and what was the least important aspect in your decision?

*Please select only one aspect in each column under "**Most important**" and "**Least important**".*

Question 7 of 13

Most Important		Least Important
	**(Item #1) Someone in my family was diagnosed with the disease**	
	**(Item #4) The time it takes for me to get results back from the genetic test**	
	**(Item #9) The genetic test results will indicate what treatment will be necessary for disease management**	
	**(Item #13) The potential of getting an inaccurate genetic test result**	

--- Page Break (Qualtrics) ---

To test or not to test? Study protocol for a best-worst scaling to understand decision-making and preferences for genetic testing in moderate-risk individuals

Thinking back to the decision you made to have (or not have) primary genetic testing: What was the most important aspect and what was the least important aspect in your decision?

*Please select only one aspect in each column under "**Most important**" and "**Least important**".*

Question 8 of 13

**Most Important**		**Least Important**
	**(Item #3) The genetic test includes an out-of-pocket fee that I must pay**	
	**(Item #4) The time it takes for me to get results back from the genetic test**	
	**(Item #7) The genetic test results will help me with family planning**	
	**(Item #8) I will need additional appointment(s) with my healthcare team and/or specialists if the genetic test results are positive**	

--- Page Break (Qualtrics) ---

Thinking back to the decision you made to have (or not have) primary genetic testing: What was the most important aspect and what was the least important aspect in your decision?

*Please select only one aspect in each column under "**Most important**" and "**Least important**".*

Question 9 of 13

**Most Important**		**Least Important**
	**(Item #5) The genetic test results will support my own future healthcare decisions**	
	**(Item #8) I will need additional appointment(s) with my healthcare team and/or specialists if the genetic test results are positive**	
	**(Item #12) Potential that someone outside of my healthcare team could access my genetic test results**	
	**(Item #13) The potential of getting an inaccurate genetic test result**	

--- Page Break (Qualtrics) ---

To test or not to test? Study protocol for a best-worst scaling to understand decision-making and preferences for genetic testing in moderate-risk individuals

Thinking back to the decision you made to have (or not have) primary genetic testing: What was the most important aspect and what was the least important aspect in your decision?

*Please select only one aspect in each column under "**Most important**" and "**Least important**".*

Question 10 of 13

**Most Important**		**Least Important**
	**(Item #2) Someone in my family had genetic testing before**	
	**(Item #6) The genetic test results will inform my family members of their risk for the disease**	
	**(Item #8) I will need additional appointment(s) with my healthcare team and/or specialists if the genetic test results are positive**	
	**(Item #9) The genetic test results will indicate what treatment will be necessary for disease management**	

--- Page Break (Qualtrics) ---

Thinking back to the decision you made to have (or not have) primary genetic testing: What was the most important aspect and what was the least important aspect in your decision?

*Please select only one aspect in each column under "**Most important**" and "**Least important**".*

Question 11 of 13

**Most Important**		**Least Important**
	**(Item #7) The genetic test results will help me with family planning**	
	**(Item #9) The genetic test results will indicate what treatment will be necessary for disease management**	
	**(Item #11) The genetic test results will provide information about risk of developing the disease and impact on life expectancy**	
	**(Item #12) Potential that someone outside of my healthcare team could access my genetic test results**	

--- Page Break (Qualtrics) ---

To test or not to test? Study protocol for a best-worst scaling to understand decision-making and preferences for genetic testing in moderate-risk individuals

Thinking back to the decision you made to have (or not have) primary genetic testing: What was the most important aspect and what was the least important aspect in your decision?

*Please select only one aspect in each column under "**Most important**" and "**Least important**".*

Question 12 of 13

Most Important		Least Important
	**(Item #2) Someone in my family had genetic testing before**	
	**(Item #3) The genetic test includes an out-of-pocket fee that I must pay**	
	**(Item #11) The genetic test results will provide information about risk of developing the disease and impact on life expectancy**	
	**(Item #13) The potential of getting an inaccurate genetic test result**	

--- Page Break (Qualtrics) ---

Thinking back to the decision you made to have (or not have) primary genetic testing: What was the most important aspect and what was the least important aspect in your decision?

*Please select only one aspect in each column under "**Most important**" and "**Least important**".*

Question 13 of 13

Most Important		Least Important
	**(Item #1) Someone in my family was diagnosed with the disease**	
	**(Item #3) The genetic test includes an out-of-pocket fee that I must pay**	
	**(Item #6) The genetic test results will inform my family members of their risk for the disease**	
	**(Item #12) Potential that someone outside of my healthcare team could access my genetic test results**	

--- Page Break (Qualtrics) ---

To test or not to test? Study protocol for a best-worst scaling to understand decision-making and preferences for genetic testing in moderate-risk individuals

Secondary Analysis

In this section of the survey, we will ask you questions about aspects that impacted your decision to have (or not have) secondary analysis in this study. **Secondary analysis includes genes that are NOT related to breast cancer or aortic diseases. However, they could provide information about your underlying risk for other diseases.**

Eighty-one (81) genes have been recommended for secondary analysis by the American College of Medical Genetics because of their “medical actionability”. This means that a person who tests positive for a disease-causing variant from secondary analysis is offered additional screening, monitoring, and/or treatment.

--- Page Break (Qualtrics) ---

1. *Please rank the following items in order of the importance they had on your decision to have (or not have) secondary analysis, with "1" meaning it was the most important item.*
   Clicking the items will show the ranking order from "1" to "6" on the left hand side. You can change the order of the ranking by holding and dragging the items to the appropriate spot.
   _ Experiencing uncertainty of not knowing about my risk for another disease
   _ Having access to my results from the secondary analysis and permission rights to decide who else can access my results outside of my healthcare team
   _ Obtaining information about another disease that I can act upon
   _ Experiencing distress or anxiety in case of receiving a positive result
   _ Obtaining information to inform my relatives about another disease
   _ Being fully informed by genetics healthcare professionals about the impacts of getting information about other potential diseases

2. *Is there anything else not listed above that impacted or changed your decision?*
   o Yes
   o No

--- Page Break (Qualtrics) ---

3. *[Filter question for those indicated choice “Yes” in Q2] If yes, please describe any other factors that impacted or changed your decision to have (or not have) secondary analysis.*
   [Free text box]

--- Page Break (Qualtrics) ---

In Alberta, the costs of some genetic tests are covered through provincial health insurance and/or some private health benefits plans. Although you had the option to have secondary analysis as part of your participation in this study, it is not currently available as a standard part of genetic testing for people who are at moderate risk.

4. ***Imagine the Government of Alberta would like to offer secondary analysis to people who are at moderate risk like you. How much would you be willing to pay out-of-pocket* for secondary analysis in CAD?***

$ [Free text – numerical only between 0 - 10 000]

Out-of-pocket *costs are the remaining costs of tests or procedures that are not covered by provincial health insurance or private health benefits plans for which patients are sometimes required to pay. For example, if a test or procedure costs $1 000, your health benefits plan may cover 80% ($800) but you would be required to pay the remaining 20% ($200). If the cost of the test or procedure is not covered at all, you would be required to pay 100% of the cost ($1 000).*

--- Page Break (Qualtrics) ---

To test or not to test? Study protocol for a best-worst scaling to understand decision-making and preferences for genetic testing in moderate-risk individuals

Experience with Genetic Testing

1. *Have you ever **heard** of genetic testing before taking part in this study?*
   - o Yes
   - o No
   - o I am not sure

--- Page Break (Qualtrics) ---

[Filter questions 1a for those indicated 'Yes' in Q1]

1a. *You indicated you have heard of genetic testing before. Which genetic tests have you **heard** of?*

[free text answer]

2. *Have you ever **had** a genetic test of any kind, including genetic screening tests, genetic tests to diagnose a disease or identify a health risk, or a genetic test to learn about your family ancestry before, taking part in this study?*
   - o Yes
   - o No
   - o I am not sure

--- Page Break (Qualtrics) ---

[Filter questions 2a-b for those indicated 'Yes' in Q2]

2a. *What types of genetic tests have you **had**? Please select all that apply.*

- □ Screening test to know your risk of future health problems
- □ Screening test when pregnant looking for the risk of health problems for the baby
- □ Diagnostic test to help find a diagnosis for a specific set of symptoms you were experiencing
- □ Diagnostic test to help pick the best treatment for a disease that you had
- □ Genetic test that was done as part of testing for a family member
- □ Genetic test to learn about family ancestry
- □ None of the above [answer exclusive]
- □ I am not sure

--- Page Break (Qualtrics) ---

2b. [Filter question for those indicated "a", "c" or "d" in Q2a] What was the result of the genetic test? Select those that apply.

- ◻ Positive (at least ONE disease-causing variant was detected)
- ◻ Negative (no disease-causing variants were detected) [answer exclusive]
- ◻ Uncertain (at least ONE variant of uncertain significance was detected)
- ◻ I am not sure / I do not remember [answer exclusive]

--- Page Break (Qualtrics) ---

3. *Please answer the following questions about what you could expect to learn from your genetic test results.*
   I would expect my genetic test results to:

	Strongly disagree	Disagree	Neither agree nor disagree	Agree	Strongly agree	*Not applicable*
Help explain a condition that I have	○	○	○	○	○	○
Help explain a family history of disease	○	○	○	○	○	○
Reassure me that I am currently healthy	○	○	○	○	○	○
Give me information about specific diseases that I am concerned about	○	○	○	○	○	○
Help tailor treatment(s) to me specifically	○	○	○	○	○	○
Help me prevent future diseases	○	○	○	○	○	○
Help me learn more about the chance of passing on a disease to my children	○	○	○	○	○	○

--- Page Break (Qualtrics) ---

4. *How much do you agree or disagree with the following statements about receiving your genetic test results?*

	Strongly disagree	Disagree	Neither agree nor disagree	Agree	Strongly agree
I am concerned about receiving genetic test results with uncertain meaning	○	○	○	○	○
I am concerned that I will not be able to understand my genetic test results	○	○	○	○	○
I am concerned about receiving information that I do not want	○	○	○	○	○
I am concerned about receiving genetic test results that I cannot do anything about	○	○	○	○	○
I am concerned about my ability to cope with receiving my genetic test results	○	○	○	○	○
I am concerned about the treatments or lifestyle changes needed to address my genetic test results	○	○	○	○	○
I am not at all concerned about receiving my genetic test results	○	○	○	○	○

--- Page Break (Qualtrics) ---

5. *Do you have any additional comments or feedback you would like to share with us?*
   [free text answer - optional]

--- Page Break (Qualtrics) ---

This is the end of the questionnaire.

Thank you for participating in our study and completing this survey!
