# Supplementary material for: To test or not to test? Study protocol for a best-worst scaling to understand decision-making and preferences for genetic testing in moderate-risk individuals
Source: PLoS One. 2025 Dec 29;20(12):e0339696. doi: 10.1371/journal.pone.0339696 (PMC12747399; doi:10.1371/journal.pone.0339696)
Supplement: S4 File — (PDF) [file pone.0339696.s004.pdf]

Supporting information: PLOS One

To test or not to test? Study protocol for a best-worst scaling to understand decision-making and preferences for genetic testing in moderate-risk individuals

Carina Oedingen[1], Nicolle Hua[1], Karen V. MacDonald[1], Julien Marcadier[2,3], Renee Perrier[2,3], Lindsay Tuer[2], Brenda McInnes[2,3], Francois Bernier[2,3], Deborah A. Marshall[1,3]

1 Department of Community Health Sciences, Cumming School of Medicine, University of Calgary, Calgary, Alberta, Canada

2 Department of Medical Genetics, Cumming School of Medicine, University of Calgary, Calgary, Alberta, Canada

3 Alberta Children's Hospital Research Institute, Calgary, Alberta, Canada

To test or not to test? Study protocol for a best-worst scaling to understand decision-making and preferences for genetic testing in moderate-risk individuals

S4. Clinical genetic testing eligibility guidelines for breast cancer

CANCER GENETICS CLINIC
Genetic Testing Guidelines

HEREDITARY BREAST OR BREAST/OVARIAN CANCER

1. Relatives of an individual with a confirmed pathogenic BRCA1 or BRCA2 mutation.

Breast Cancer

1. Personal history of breast cancer diagnosed ≤ 35.§.
2. Personal history of two primary breast cancers; one diagnosed under ≤ 50 OR both diagnosed ≤ age 60.§
3. Personal history of breast and ovarian cancer*.§.
4. Personal history of breast and pancreatic cancer.§
5. Personal history of breast cancer ≤ 50 AND a family history of breast cancer ≤ 50.
6. Personal history of breast cancer AND family history of ovarian cancer* diagnosed at any age.
7. Personal history of breast cancer AND two family members with breast cancer; one diagnosis ≤ 50
8. Personal history of breast cancer AND two family members with pancreatic adenocarcinoma at any age.
9. Personal history of triple negative breast cancer (ER-ve, PR-ve, Her2-ve) diagnosed ≤ age 65.§.
10. Personal history of male breast cancer diagnosed at any age.§.
11. Personal history of breast cancer and family history of male breast cancer.
12. Personal history of breast cancer at any age and a first degree relative meeting a "§"criterion.

Ovarian Cancer

1. Personal history of invasive epithelial ovarian/fallopian tube/primary peritoneal cancer at any age.

Pancreatic Cancer

1. Personal history of pancreatic adenocarcinoma at any age AND 2 or more close relatives with breast/ovarian/pancreatic cancer at any age.

Ashkenazi Jewish Families

1. Personal history of breast or ovarian cancer* at any age (testing limited to the Ashkenazi Jewish mutation panel and only followed by a full screen for individuals who meet one of the above criteria).
2. Unaffected individuals with a first or second-degree relative with breast/ovarian cancer* at any age (testing limited to the Ashkenazi Jewish mutation panel).

*Ovarian cancer = invasive epithelial ovarian cancer, and includes primary peritoneal cancers and primary fallopian tube cancers.
§ = Mainstreaming criteria; patient meeting these criteria can have genetic testing ordered through approved surgeon/oncologist.

Family history includes first, second, and third degree relatives
